# Sub-lethal effects of permethrin exposure on a passerine: implications for managing ectoparasites in wild bird nests

**DOI:** 10.1093/conphys/coaa076

**Published:** 2020-09-08

**Authors:** Mariana Bulgarella, Sarah A Knutie, Margaret A Voss, Francesca Cunninghame, Brittany J Florence-Bennett, Gemma Robson, Robert A Keyzers, Lauren M Taylor, Philip J Lester, George E Heimpel, Charlotte E Causton

**Affiliations:** 1 School of Biological Sciences, Victoria University of Wellington, PO Box 600, Wellington, 6140, New Zealand; 2 Department of Ecology and Evolutionary Biology, University of Connecticut, 75 N. Eagleville Road, Unit 3043, Storrs, CT, 06269, USA; 3 Syracuse University, Syracuse, NY, 13244, USA; 4 Charles Darwin Research Station, Charles Darwin Foundation, Puerto Ayora, Santa Cruz Island, Galápagos Islands, Ecuador; 5 School of Chemical and Physical Sciences, Victoria University of Wellington, PO Box 600, Wellington, 6140, New Zealand; 6 Department of Entomology, University of Minnesota, 1980 Folwell Avenue, St. Paul, MN, 55108, USA

**Keywords:** Birds, nest parasites, permethrin, reproductive success, zebra finch

## Abstract

Permethrin is increasingly used for parasite control in bird nests, including nests of threatened passerines. We present the first formal evaluation of the effects of continued permethrin exposure on the reproductive success and liver function of a passerine, the zebra finch (*Taeniopygia guttata*), for two generations. We experimentally treated all nest material with a 1% permethrin solution or a water control and provided the material to breeding finches for nest building. The success of two consecutive clutches produced by the parental generation and one clutch produced by first-generation birds were tracked. Finches in the first generation were able to reproduce and fledge offspring after permethrin exposure, ruling out infertility. Permethrin treatment had no statistically significant effect on the number of eggs laid, number of days from clutch initiation to hatching, egg hatch rate, fledgling mass or nestling sex ratio in either generation. However, treating nest material with permethrin significantly increased the number of hatchlings in the first generation and decreased fledgling success in the second generation. Body mass for hatchlings exposed to permethrin was lower than for control hatchlings in both generations, but only statistically significant for the second generation. For both generations, an interaction between permethrin treatment and age significantly affected nestling growth. Permethrin treatment had no effect on liver function for any generation. Permethrin was detected inside 6 of 21 exposed, non-embryonated eggs (28.5% incidence; range: 693–4781 ng of permethrin per gram of dry egg mass). Overall, results from exposing adults, eggs and nestlings across generations to permethrin-treated nest material suggest negative effects on finch breeding success, but not on liver function. For threatened bird conservation, the judicious application of this insecticide to control parasites in nests can result in lower nestling mortality compared to when no treatment is applied. Thus, permethrin treatment benefits may outweigh its sub-lethal effects.

## Introduction

Pyrethroid insecticides, such as permethrin, are synthetic analogues of pyrethrin that is produced from the flowers of *Chrysanthemum cinerariaefolium* (Palmquist *et al*., 2012). After the ban on organochlorine insecticides in the 1980s, pyrethroids became widely used (Moore *et al*., 2009). Although pyrethroids are highly toxic to fish, tadpoles and aquatic invertebrates, affecting their skin touch receptors and balance organs (Tomlin, 1994), they are considered practically non-toxic to birds and mammals due to their rapid metabolization (Demoute, 1989; Mineau *et al*., 2001; Meléndez *et al*., 2006). Despite pyrethroids having somewhat low environmental persistence of <90 days (UH, 2011), they are ubiquitous in the environment due to their continuous use (Corcellas *et al*., 2017). Permethrin is a pyrethroid first marketed in 1977 mostly for agricultural purposes (World Health Organization, 1990; Pap *et al*., 1996). It is also widely used as a home and garden insecticide, on pets and livestock, for mosquito control and for the treatment of ectoparasitic diseases, among others (Fishel, 2005).

For some threatened bird species, the management of parasites is an essential component of recovery plans because of their impact on reproductive success (Bulgarella *et al.*, 2019). For example, in the Dominican Republic, nest parasitism by the fly *Philornis pici* is a contributing factor to the decline of the critically endangered Ridgway’s hawk (*Buteo ridgwayi*; BirdLife International, 2018; Hayes *et al*., 2019). Fledgling numbers of Ridgway’s hawk have increased following applications of the insecticide permethrin to nests and the consequent reduction of *P. pici* larvae (M. Quiroga, *pers. comm*.). In Tasmania, the forty-spotted pardalote (*Pardalotus quadragintus*) is an endangered (BirdLife International, 2016) and declining passerine and parasitism by the fly *Passeromyia longicornis* is the main cause of nestling mortality. Currently, treating pardalote nests with permethrin in combination with piperonyl butoxide and methoprene is the only effective method available to boost reproductive output (Edworthy *et al*., 2019). Permethrin is also used for experimentally manipulating ectoparasite load in nests for research purposes. Species whose nests have been treated with permethrin include purple martins (*Progne subis*; Moss and Camin, 1970), cliff swallows (*Hirundo pyrrhonota*; Brown and Brown, 1986), barn swallows (*Hirundo rustica*; Møller, 1990), blue tits (*Cyanistes caeruleus*; Tomás *et al*., 2007; Lobato *et al*., 2008), pied flycatchers (*Ficedula hypoleuca*; Lobato *et al*., 2008), red grouse (*Lagopus lagopus*; Mougeot *et al*., 2008), tropical mockingbirds (*Mimus gilvus*; Knutie *et al*., 2017), black-faced grassquits (*Tiaris bicolor*; Knutie *et al*., 2017) and barn owls (*Tyto alba*; Efstathion *et al*., 2019).

Recently, permethrin was selected as the best insecticide candidate to treat nests of land bird species in the Galapagos Islands (Causton and Lincango, 2014) as a stop-gap control measure against an invasive nest parasite, the muscid fly *Philornis downsi*. This parasite is the main cause of bird population declines in the archipelago, particularly affecting Darwin’s finches and other passerines (Kleindorfer and Dudaniec, 2016; Fessl *et al*., 2018; McNew and Clayton, 2018). *Philornis downsi* is an obligate bird parasite introduced to the Galapagos Islands before or during the 1960s (Causton *et al*., 2006). Adult female flies lay eggs within active bird nests, and the larvae feed on the blood and serous fluids of nestlings. Larval feeding by *P. downsi* can cause anemia and beak scarring/deformation in host birds and can lead to high mortality of infested nestlings (reviewed in Kleindorfer and Dudaniec, 2016; Fessl *et al*., 2018). A number of potential long-term control methods for *P. downsi* are being studied including biological control and sterile male release (reviewed in Fessl *et al*., 2018). In the meantime, short-term solutions are essential to protect Darwin’s finches and other land birds in serious, rapid decline (Fessl *et al*., 2017). The best option currently available is the injection of permethrin into the base of nests where the larvae are located. However, this method poses several challenges due to the inaccessibility of many nests (Causton *et al*., 2019). One solution to this problem is a self-fumigation technique developed by Knutie *et al*. (2014), who treated cotton fibres with a 1% solution of permethrin and offered the cotton to Darwin’s finches at a field site in Galapagos. The researchers found that four of the more common finch species readily incorporated cotton fibres into their nests, which dramatically reduced the number of *P. downsi* larvae. This result has been widely recognized as a breakthrough in potential methods to protect endangered bird populations from ectoparasites (Morrison, 2014; Williams, 2014). However, this particular use of permethrin requires further evaluation to ensure safety to endangered passerine species.

Permethrin has been used experimentally in nests of Galapagos birds to evaluate the effect of *P. downsi* on reproductive success in at least five studies, all of which resulted in higher nestling survival (Koop *et al*., 2013a,b; Knutie *et al*., 2013, 2016; O’Connor *et al*., 2014). This suggests that low doses (1% aqueous solution) of this insecticide do not have lethal effects on passerine birds or noticeable sub-lethal effects on reproductive success when used for short periods of time (exposure time is restricted to the days that nestlings spend in the nest, i.e. from hatching until fledging). These results are similar to other studies using a variety of bird species that did not report adverse effects of permethrin on bird reproductive output and have found that fledgling success was often higher in nests treated with low doses of permethrin compared to untreated, parasitized nests. It is worth noting that the increase in fledging success was highly likely due to the removal of parasites, as opposed to any potential benefits of permethrin exposure (e.g. Moss and Camin, 1970; Brown and Brown, 1986; Møller, 1990; Pap *et al*., 2005; Tomás *et al*., 2007; Lobato *et al*., 2008; Efstathion, 2015; Efstathion *et al*., 2019). However, the objective of the above-mentioned studies was to understand the effects of ectoparasites on birds and not to study the effects of permethrin itself.

Most of the experimental studies that treated bird nests with permethrin compared insecticide-treated nests versus nests infested by ectoparasites, some of which have deleterious effects on birds (reviewed in Causton and Lincango, 2014). Using parasitized nests as controls makes it hard to discern any effects that permethrin might have on bird body condition and reproductive success. An exception is a study by López-Arrabé *et al.* (2014) who tested the effects of using a combination of permethrin, tetramethrin and the synergist piperonyl butoxide on pied flycatcher nesting success versus treating nests with heat or no treatment at all. The authors found negative effects of the insecticide mix on nestling growth and differences in levels of total glutathione, a biomarker associated with cellular detoxification. We are not aware of any studies that tested the sub-lethal effects of permethrin by itself on the reproductive success of small bird species or of studies that have looked at the effects of permethrin over consecutive generations.

The objective of our study was to determine the effects of permethrin exposure on reproductive success, fertility and liver function of zebra finches (*Taeniopygia guttata*) across two consecutive generations under controlled laboratory conditions. We experimentally treated all potential nest material with either a 1% permethrin solution or a water control, then provided the material to nesting finches to build their nests. We also determined if permethrin was detected inside unhatched, non-embryonated finch eggs.

## Materials and methods

### Experimental birds

Zebra finches are a representative study species because their size, incubation period and clutch size are similar to that of the majority of Darwin’s finches (Grant and Grant, 1980; Zann, 1996), both belong to the superfamily Passeroidea within the order Passeriformes, and much information already exists about their physiology, behaviour and genetics (Fritz *et al*., 2014). We acquired the female zebra finches from a local breeder in Wellington, New Zealand, and the male finches from a pet store in Auckland, New Zealand. The initial colony consisted of 20 zebra finch pairs, the parental generation, aged between 7 months and 2 years old when the study started. Finches did not have previous experience building a nest. Each bird received a plastic, numbered, split leg band for individual identification. Birds were kept on a 14:10 light:dark cycle in a room equipped with full-spectrum light tubes (Viva-Lite, Germany; CRI 96–98, colour temperature: 5500 K), at a temperature of 23°C (range: 20–26°C) and relative humidity of 50% (range: 30–69%). Birds were provided with food, tap water and cuttlefish *ad libitum*, as well as water dishes for bathing. Food consisted of finch mix (canary seed, panicum, white French millet, red panicum, Japanese millet, oilseed rape and linseed; TopFlite Ltd, Oamaru, New Zealand). Finches were housed in pairs in wire cages measuring 34 × 26.5 × 51 cm (Avi One model 320 H, Kong’s NZ Ltd, New Zealand). Breeding pairs were provided with crushed hard-boiled egg or premium egg and biscuit food (MasterPet, Lower Hutt, New Zealand) once a week and organic spinach once every 2 weeks. Vitamins were added to the drinking water once a month (VetaFarm Multivet; NSW, Australia). All bird husbandry and experimental protocols were approved by the Animal Ethics Committee of Victoria University of Wellington (protocol 22775).

### Experimental design

**Figure 1:**
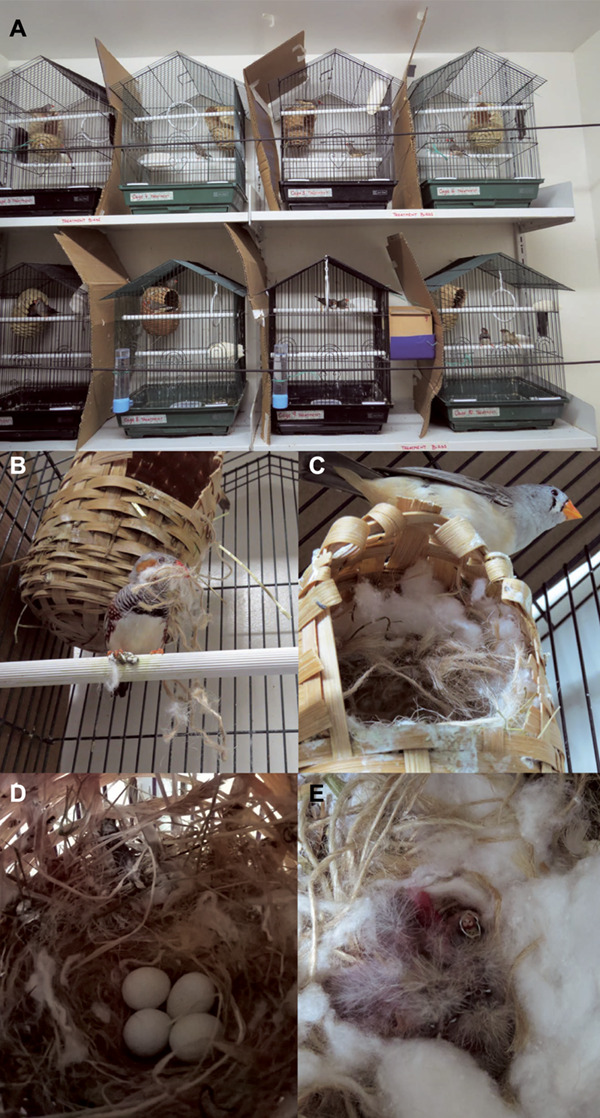
Experimental set-up. (A) Arrangement of cages in the laboratory, (B) male zebra finch (*Taeniopygia guttata*) carrying treated nest materials to its nest basket, (C) female zebra finch on top of its nest basket, (D) eggs resting on permethrin-treated nest materials, (E) nestlings resting on permethrin-treated nest materials.

Our experiment consisted of two treatments. We randomly assigned the birds to either treatment so that 10 finch pairs formed the experimental group and 10 pairs the control group. Pairs were established at random. Each cage was fitted with a bamboo nest basket (12 × 15 cm, Trixie, Germany). Cardboard separated adjacent cages so that bird pairs could not see neighbour pairs but they may have seen cages across the room (Fig. 1A; Muth and Healy, 2011). Nesting material consisted of organic lucerne hay (*Medicago sativa*; MasterPet, Lower Hutt, New Zealand), sisal fibre consisting of a mixture of sisal, jute and cotton (Best Bird, New Plymouth, New Zealand) and cotton wool. The two treatments consisted of providing daily (1) ~5 g of the above-mentioned mixed nesting materials that had been sprayed with ~3 ml of 1% solution of permethrin per 30 cm^2^ (Permectrin II, Bayer, Shawnee, KS, USA) (experimental group) and (2) ~5 g of mixed nesting materials that had been sham-fumigated with water (control group). Nesting material was provided for ~10–14 days, which included the nest building and egg laying phases. We ceased to provide nest materials when a clutch was complete to avoid ‘sandwich clutches’ (Nager and Law, 2010). Every piece of material that the experimental group received was permethrin treated; therefore, our design simulated a scenario in which the adult birds were continually manipulating the treated material with their beaks, and incubating eggs and nestlings rested upon treated materials (Fig. 1). The numbers of eggs in the nest baskets were recorded daily. The number of days from clutch initiation to hatching was calculated in days by subtracting the date when the first egg was laid to the date when the first chick hatched. Once nestlings hatched, each sibling was marked on the tarsometatarsus with a non-toxic marker (Sharpie®, Newell Rubbermaid, Oak Brook, IL, USA) of a different colour for identification and weighed daily to the nearest 0.001 g using an MPB precision scale (Millennium Mechatronics, Auckland, New Zealand) until 15 days post-hatch. We considered the body mass at age 15 days post-hatch to be the fledgling mass. Fledglings were kept in the cage with their parents until roosting independence at 50 days post-hatch (Zann, 1996). Then, the fledglings were transferred to shared ‘F1.1 control’ or ‘F1.1 experimental’ wire group cages (60 × 41.5 × 41.5 cm, Avi One) until sexual maturity (~90 days post-hatch; Zann, 1996) without access to nesting materials. The parental generation laid a second clutch of eggs (F1.2) using treated or untreated nesting materials, as previously described. When the parental generation had fledged two broods of birds, they were removed from the study. The nestlings from the F1.1 generation were paired with non-sibling birds in their same treatment group, and they fledged one brood of nestlings (F2). The numbers of eggs were recorded daily, and hatchlings were marked and weighed as described above for the F1 generation. The F1.2 and F2 generations were exposed to permethrin-treated materials if they were in the treatment group or sham-fumigated materials if they were control birds. Fledglings in the F1.2 and F2 generations were weighed and bled when they reached 6 weeks of age and subsequently removed from the study.

### Detection of permethrin in eggs

#### Egg sampling

Non-embryonated eggs were collected after determining status by egg candling. We selected non-embryonated eggs because it is recommended not to mix eggs with and without embryonic development in chemical analyses as these can differ significantly in the concentration of analytes (Orłowski *et al*., 2016). The eggs were removed from the nests and frozen at −20°C in individually labelled bags. A total of 49 samples corresponding to 13 control eggs spiked with permethrin as a standard (see below), 15 eggs from the control group and 21 eggs from the experimental group were collected and the eggshell removed. The egg content was weighed on a Mettler Toledo AG204 analytical balance (Mettler Toledo Ltd, Hamilton, New Zealand), homogenized and freeze-dried overnight. Lyophilized samples were weighed and homogenized again and stored at −20°C until further analyses.

#### Analytical methodologies

Sample treatment followed Corcellas *et al*. (2017). An exact amount between 0.1 g and 0.2 g of freeze-dried egg mass was used. For the 13 spiked eggs, egg mass was spiked overnight with 1.25 μg (10 μl of 31.5 mM solution) of phenoxy-d_5_-cis-permethrin (Sigma Aldrich, New Zealand) as an internal standard. For all egg samples, standard solutions were prepared in ethyl acetate (gas chromatography grade, Merck, New Zealand) while sample mixing was achieved using a vortex mixer. Calibration curves were then prepared at different concentrations ranging between 0.01 μg ml^−1^ and 20 μg ml^−1^ total permethrin (EPA test standard, mixture of *cis* and *trans* isomers, 1000 μg/ml total, Supelco, Bellefonte, PA, USA). Solid-phase extraction (SPE) cartridges (Agilent Bond Elut C18 OH, 500 mg, 3 ml; Agilent Bond Elut AL-B 500 mg, 3 ml) were obtained from DKSH Ltd (New Zealand).

For all egg samples (13 spiked, 15 control and 21 treatment eggs), the permethrin extraction procedure was carried out twice with 20 ml of hexane:dichloromethane 2:1 and assisted by ultrasonication for 15 min. All solvent was left to evaporate in a fumehood overnight. A tandem SPE clean up (basic alumina and C18 cartridges, 30 ml acetonitrile as eluent) was subsequently carried out. The eluent was evaporated under N_2_ with a sample concentrator, then reconstituted in 100 μl of ethyl acetate. Analyses were performed on a Shimadzu QP-2010 gas chromatograph coupled to a QP-2010 Plus electron impact mass spectrometer (Kyoto, Japan). The gas chromatograph–mass spectrometer was equipped with a Restek Rxi-5SilMS column (30 m × 0.25 mm × 0.25 μm; Bellefonte, PA, USA) using ‘zero-grade’ He as a carrier gas at a flow rate of 1.43 ml/min (43.4 cm/s linear velocity). Samples were injected (1 μl; splitless injection) in a split/splitless liner held at 270°C. The MS interface line was held at 305°C while the EI-MS (70 eV) source was held at 200°C. The column oven temperature program was as follows: begin at 50°C and hold for 2 mins, ramp at 15°C to 300°C and hold for 5 mins. Mass spectra were acquired every 0.3 s over the range *m/z* 42–600. Extracted ion chromatograms were integrated to measure permethrin content (quantitative ion: *m/z* 153; qualitative ion: *m/z* 183; internal standard ion: *m/z* 188) in chromatograms. A quantitative:qualitative ion ratio of 0.05–0.20 was required for positive detection, results outside of this ratio were discounted.

#### Quality assurance/control

Linearity in the selected range of concentrations was verified by obtaining a correlation coefficient higher than 0.99 for total permethrin content. Using the spiked egg samples, 5 of the 13 samples did not return measurable permethrin above the limit of detection (LOD). For the remaining 8 samples, the mean recovery was 85.5%, with a range of 74.9–107.0% (standard deviation, 3.8%). The total permethrin LOD was 4.5 ng per 0.1 g of dried egg mass, and the limit of quantification (LOQ) was 11.5 ng total permethrin per 0.1 g of dried egg mass. Detections between the LOD and LOQ are described as trace amounts.

### Blood draw protocol for liver function tests

Blood samples (50 μl) were collected into heparinized microcapillary tubes (Drummond Scientific Company, Broomall, PA, USA) via brachial venipuncture from each bird. Whole blood was centrifuged for 5 mins at 14 000 g to separate the plasma from the blood cells. The plasma from 4 to 5 birds (within the same treatment group, and of the same sex and clutch number) was pooled to reach the minimum volume (200 μl) required for quantification. Plasma was stored at −80°C until it was sent to New Zealand Veterinary Pathology (Hamilton, New Zealand) for analyses.

Blood samples were drawn from the parental generation 3 times. The first sample was taken before experimental treatments began (baseline bleeding; *n* = 40 birds). The baseline blood draw took place on 7 and 8 January 2017. The second blood draw took place after the birds had access and were exposed to treated nest material for at least 4 days. The third blood draw took place after the F1.1 nestlings fledged, after ~70 days of exposure to treated materials. For the F1.1 generation, bleeding took place when the birds reached 6 weeks of age as that is when their immune system is fully developed (Schmidt, 1997; Wakenell, 1999). A second blood sample was drawn from the F1.1 generation during the nest building or incubation phase after the birds had manipulated the nest material for at least 4 days. We bled the F1.2 generation nestlings when they reached 6 weeks of age.

#### Methodology for blood plasma analyses

The plasma levels of two enzymes, aspartate aminotransferase (AST) and creatinine kinase (CK), and one metabolite class, bile acids (BA), were assayed in this study. Hereafter, we consider the levels of AST and BA as indicative of liver function. All three analytes were run in a Beckman Coulter AU680 Clinical Chemistry Analyser (Brea, CA, USA) at New Zealand Veterinary Pathology (Hamilton, New Zealand).

Aspartate aminotransferase catalyses the transamination of aspartate and 2-oxoglutarate, forming L-glutamate and oxalacetate. Pyridoxal phosphate was added to the reaction mixture to ensure maximum catalytic activity of AST. The oxalacetate is reduced to L-malate by malate dehydrogenase, while reduced nicotinamide adenine dinucleotide (NADH) is simultaneously converted to NAD+. The decrease in absorbance due to the consumption of NADH is measured at 340 nm and is proportional to the AST activity in the sample. Endogenous pyruvate is removed by the lactate dehydrogenase reaction during the incubation period (Beckman Coulter reagent OSR6009, New Zealand).

Bile acid levels in the plasma samples were determined using an enzymatic method. In the presence of Thio-NAD, the enzyme 3-a hydroxysteroid dehydrogenase (3-αHSD) converts BAs to 3-keto steroids and Thio-NADH. The reaction is reversible and 3-αHSD can convert 3-ketosteroids and Thio-NADH to BAs and Thio-NAD. In the presence of excess NADH, the enzyme cycling occurs efficiently and the rate of formation of Thio-NADH is determined by measuring the specific change of absorbance at 405 nm.

Plasma levels of CK activity were determined by activation with N-acetylcysteine. Creatinine kinase reversibly catalyses the transfer of a phosphate group from creatinine phosphate to adenosine diphosphate (ADP) to give creatinine and adenosine triphosphate (ATP) as products. The ATP formed is used to produce glucose-6-phosphate and ADP from glucose. This reaction is catalysed by hexokinase, which requires magnesium ions for maximum activity. The glucose-6-phosphate is oxidized by the action of the enzyme glucose-6-phosphate dehydrogenase with simultaneous reduction of the coenzyme NADH to give nicotinamide adenine dinucleotide phosphate (NADPH) and 6-phosphogluconate. The rate of increase of absorbance at 340/660 nm due to the formation of NADPH is directly proportional to the activity of CK in the plasma sample (Beckman Coulter reagent OSR6279, New Zealand).

### Statistical analyses

For the first generation, generalized linear mixed models (GLMM) with the variable ‘cage’ as a random effect were used to determine the effect of treatment (permethrin or no permethrin), clutch number (first or second clutch) and their interaction on the number of eggs laid, the number of days from clutch initiation to hatching, the number of nestlings that hatched, the number of nestlings that fledged and hatchling and fledgling body mass (while controlling for brood size). Binomial GLMMs (i.e. logistic regression) with the variable ‘cage’ as a random effect were also used to determine the effect of treatment, clutch number and their interaction on the proportion of eggs that hatched, nestling sex ratio and the proportion of nestlings that fledged (calculated as the number of nestlings that fledged/the number of eggs that hatched).

For the second generation, generalized linear models (GLM) were used to determine the effect of treatment on the number of eggs laid, the number of days from clutch initiation to hatching and the number of nestlings. GLMMs with the variable ‘cage’ as a random effect were used to determine the effect of treatment on hatchling and fledgling body mass (while controlling for brood size). Binomial GLMs were also used to determine the effect of treatment on the proportion of eggs hatched, nestling sex ratio and proportion of nestlings fledged.

Analyses were conducted using the glm (GLM) and glmer or lmer (GLMM) functions with the lme4 package (Bates *et al*., 2015). Probability values were calculated using log-likelihood ratio tests using the analysis of variance (ANOVA) function in the car package (Fox and Weisberg, 2019). Analyses were conducted in R v.3.4.3 (R Core Team, 2017) and figures were created in GraphPad Prism v.7 (GraphPad Software, La Jolla, CA, USA).

For analyses of liver function tests, blood biomarker (AST, BA and CK) values were log transformed because the plasma concentrations of the three biomarkers were not normally distributed. The data were checked for outliers using Mahalanobis distances and further tested for non-linearity and multicollinearity. Levene’s test (Levene, 1960) and Box’s test (Box, 1949) were used to confirm homogeneity of variance and covariance matrices, respectively. Assumptions of homogeneity of variances and equality of covariance matrices were met for all analyses. An *a priori* power analysis was conducted to verify if sample sizes were large enough to detect a difference between means based on the variance. For the parental generation, a complete and balanced set of blood samples were obtained. A GLM with a split-plot repeated-measures analysis was used to test for differences in AST, BA and CK levels between treatment groups (permethrin and a no permethrin control) and sexes at 3 points in time (baseline, 4 days after nest material exposure, and 70 days post-exposure). A GLMM analysis with a compound symmetry covariance structure and restricted maximum likelihood estimation was used to determine the effect of the permethrin treatment on biomarker changes between 6 weeks of age and sexual maturity in the F1.1 generation; the variable ‘cage’ was treated as a random ‘subject’ effect for this analysis, with fixed effects of sample time, sex and permethrin treatment. Nestlings were sampled at 6 weeks of age and again at sexual maturity after manipulation of treated nest material. Blood samples were pooled according to treatment group, sample time and sex to provide enough plasma for biomarker analysis; thus, the variable ‘cage’ was the experimental unit upon which repeated observations were made, as opposed to the individual bird. Samples from individual nestlings were not used for more than one pooled analysis. We lack a complete set of comparable measurements for the F1.2 generation, so F1.2 nestlings were excluded from this analysis.

We were able to obtain blood samples (pooled by cage, treatment and sex) for the F1.2 generation at only one time point (6 weeks of age). A multivariate analysis of variance (treatment × generation × sex with cage as the experimental unit) was used to compare the blood biomarker values of the F1.1 and F1.2 nestlings at 6 weeks of age. Our sample sizes were small, and some measurements were missing; therefore, Pillai’s trace statistic and an α = 0.01 were used to evaluate statistical significance conservatively (Tabachnick and Fidell, 2007). Blood biomarker analyses were done in SPSS Statistics for Windows v.25.0 (IBM Corp., Armonk, NY, USA). Values presented correspond to means ± SE unless otherwise specified.

## Results

### Effects of permethrin on bird reproduction

#### F1 generation

**Figure 2:**
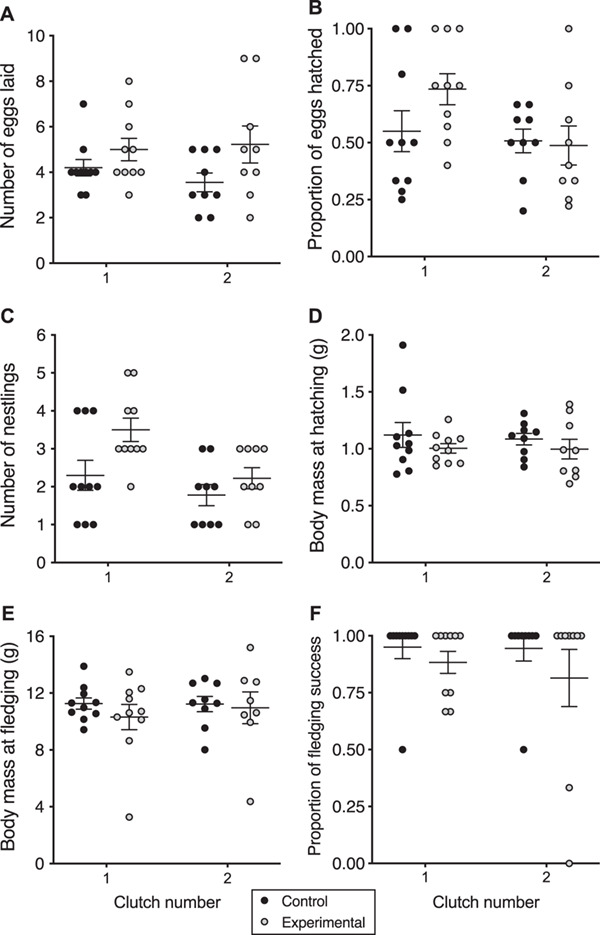
Reproductive variables measured for the first generation (F1) of zebra finches compared by clutch number: F1.1, first brood from the parental generation, and F1.2, second brood from the parental generation. (A) Number of eggs laid, (B) proportion of eggs laid that hatched, (C) number of nestlings at hatch, (D) nestling body mass at day 0, (E) nestling body mass at day 15, (F) fledgling success. Because nestlings within a nest are not independent data for body mass (D, E) are averaged over a nest so that each nest is a data point. The bars represent the overall mean ± standard error.

For the parental generation, each treatment consisted of 10 bird pairs. While there was a trend for females in the permethrin group to lay more eggs than females in the control group, this effect was not statistically significant (*χ^2^* = 3.02, *df* = 1, *P* = 0.08). Clutch number did not significantly affect the number of eggs laid (Fig. 2A; *χ^2^* = 0.40, *df* = 1, *P* = 0.53), and the interaction between treatment and clutch number did not significantly affect the number of eggs laid (*χ^2^* = 0.85, *df* = 1, *P* = 0.36).

The number of days from clutch initiation to hatching was not significantly affected by permethrin treatment (*χ^2^* = 0.31, *df* = 1, *P* = 0.58), clutch number (*χ^2^* = 0.24, *df* = 1, *P* = 0.63) or the interaction between treatment and clutch number (*χ^2^* = 0.00, *df* = 1, *P* = 0.99).

Treatment also did not significantly affect the proportion of eggs hatched (*χ^2^* = 0.39, *df* = 1, *P* = 0.53). However, the first clutch had a significantly higher proportion of eggs hatch than did the second clutch (Fig. 2B; *χ^2^* = 5.08, *df* = 1, *P* = 0.02). The interaction between treatment and clutch number did not significantly affect the proportion of eggs hatched (*χ^2^* = 2.26, *df* = 1, *P* = 0.13). The egg failure rate observed for the first clutch (F1.1) of control group finches was 45%, whereas for the permethrin-treated group, it was 27%. For the second clutch (F1.2), we recorded a 49% egg failure rate for the control group and 56% for the permethrin-exposed finches. Table S1 summarizes clutch sizes and mean egg hatch rates for the control and permethrin treatment groups.

Finches in the permethrin treatment produced significantly larger broods (i.e. the number of nestlings at hatch) than finches in the control group (*χ^2^* = 6.80, *df* = 1, *P* = 0.009). Overall brood size at hatching was significantly smaller for the second clutch than for the first clutch (Fig. 2C; *χ^2^* = 7.74, *df* = 1, *P* = 0.005), but the interaction between treatment and clutch number did not affect brood size (*χ^2^* = 1.36, *df* = 1, *P* = 0.24). Neither permethrin treatment nor clutch number significantly affected the sex ratio of the F1 nestlings (treatment: *χ^2^* = 0.28, *df* = 1, *P* = 0.60; clutch number: *χ^2^* = 0.06, *df* = 1, *P* = 0.81). The interaction between group and clutch number did not significantly affect sex ratio (*χ^2^* = 2.71, *df* = 1, *P* = 0.10).

We found that permethrin treatment had a marginally non-significant effect on hatchling mass (mass at day 0; Fig. 2D; *χ^2^* = 3.68, *df* = 1, *P* = 0.06) with higher mass at day 0 in control nestlings compared to permethrin-treated nestlings. However, clutch number (*χ^2^* = 0.02, *df* = 1, *P* = 0.88) and the interaction between treatment and clutch number (*χ^2^* = 0.66, *df* = 1, *P* = 0.42) did not significantly affect hatchling mass.

Fledging mass (mass at day 15) was also not significantly affected by treatment (*χ^2^* = 0.37, *df* = 1, *P* = 0.54), clutch number (Fig. 2E; *χ^2^* = 2.00, *df* = 1, *P* = 0.16) or the interaction between treatment and clutch number (*χ^2^* = 1.24, *df* = 1, *P* = 0.27). Clutch number significantly affected the total number of fledglings (*χ^2^* = 8.16, *df* = 1, *P* = 0.004); across treatments, the first clutch produced more fledglings than the second clutch. However, permethrin treatment (*χ^2^* = 1.63, *df* = 1, *P* = 0.20) and the interaction between treatment and clutch number (*χ^2^* = 1.79, *df* = 1, *P* = 0.18) did not significantly affect the total number of fledglings produced per nest. Additionally, treatment (Fig. 2F; *χ^2^* = 2.58, *df* = 1, *P* = 0.11), clutch number (*χ^2^* = 1.53, *df* = 1, *P* = 0.22) and the interaction between treatment and clutch number (*χ^2^* = 0.11, *df* = 1, *P* = 0.74) did not significantly affect the proportion of fledglings out of the total number of nestlings (fledgling success).

**Figure 3:**
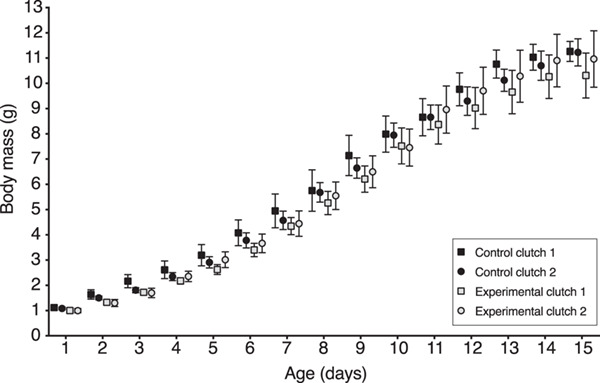
Nestling body mass gain from day hatched until fledging for the F1 generation of zebra finches comparing control versus permethrin-treated groups by clutch. Data for body mass are presented as a mean per nest. Error bars correspond to standard errors.

Permethrin treatment alone (*χ^2^* = 0.28, *df* = 1, *P* = 0.60) did not significantly affect nestling growth. As expected, nestlings gained mass as they grew older (*χ^2^* = 8162.31, *df* = 1, *P* < 0.0001). However, the interaction between treatment and nestling age significantly affected nestling growth (Fig. 3; *χ^2^* = 9.97, *df* = 1, *P* = 0.002) where the effect of permethrin treatment on growth depended on nestling age. The interactions between treatment and clutch number (*χ^2^* = 0.73, *df* = 1, *P* = 0.39), between clutch number and age (*χ^2^* = 1.55, *df* = 1, *P* = 0.21) and among treatment, clutch number and age (*χ^2^* = 0.24, *df* = 1, *P* = 0.62) did not significantly affect nestling growth.

One parental pair in cage 4 of the permethrin-treated group laid and hatched 3 eggs in their first clutch. The nestlings’ growth was extremely slow, compared to other treatment cages and by the age of 15 days, the 3 nestlings weighed: 3.9, 3.1 and 2.8 g, respectively, compared to the weight of a normally developed nestling of ~ 9–13 g at age 15 days (see outlier in first clutch of Fig. 2E). All 3 nestlings had eyes closed at 15 days old, which is not the norm. Nestling 2 and 3 died at ages 27 and 20 days, respectively. While nestling 1 survived, it weighed only 10.1 g at 59 days of age. The nestling that survived was a male, and it was crossed with 3 different females from the F1.1 generation but failed to fertilize any eggs. These females were subsequently crossed with different males and they laid fertile eggs.

Another case of underdeveloped nestlings was observed in the second clutch of the parental pair in cage 9 of the permethrin-treatment group. In this case, the finch pair successfully fledged 2 nestlings in their first brood. For their second brood, 3 nestlings hatched but only 1 survived to 15 days old. This individual weighed 4.4 g and had not developed body feathers by age 15 days (see outlier in second clutch of Fig. 2E).

#### F2 generation

**Figure 4:**
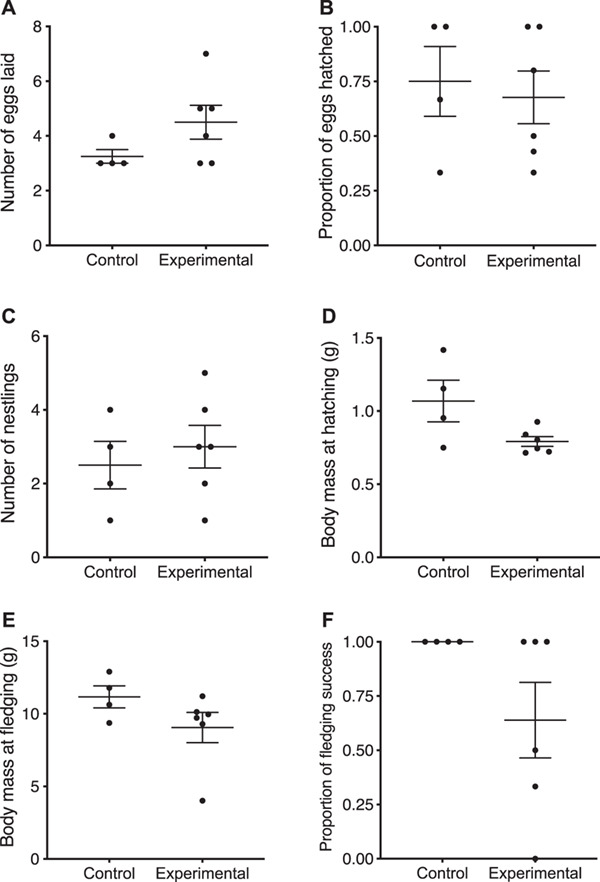
Reproductive variables measured for the second generation (F2) of zebra finches comparing control (*n* = 4 pairs) versus permethrin-treated nests (*n* = 6 pairs). (A) Number of eggs laid, (B) proportion of eggs that hatched, (C) number of nestlings at hatch, (D) nestling body mass at day 0, (E) nestling body mass at day 15, (F) fledging success. Because nestlings within a nest are not independent data for body mass (D, E) are averaged over a nest so that each nest is a data point. The bars represent the overall mean ± standard error.

The second generation consisted of the crosses of 6 pairs of birds from the permethrin-treated group and 4 from the control group, descended from the F1.1 generation. The second generation only produced 1 clutch (due to the experiment being terminated). Permethrin treatment did not affect the number of eggs laid (Fig. 4A; *χ^2^* = 2.45, *df* = 1, *P* = 0.12), the proportion of eggs that hatched (Fig. 4B, Table S1; *χ^2^* = 0.45, *df* = 1, *P* = 0.50), brood size at hatching (Fig. 4C; *χ^2^* = 0.32, *df* = 1, *P* = 0.57), the number of days from clutch initiation to hatching (*χ^2^* = 0.63, *df* = 1, *P* = 0.43) or nestling sex ratio (*χ^2^* = 0.74, *df* = 1, *P* = 0.39). Hatchling body mass was significantly higher for the control group than for the experimental group hatchlings (Fig. 4D; *χ^2^* = 14.87, *df* = 1, *P* = 0.0001). Permethrin treatment did not significantly affect fledgling mass (Fig. 4E; *χ^2^* = 1.66, *df* = 1, *P* = 0.20). Permethrin treatment affected fledgling success (Fig. 4F; *χ^2^* = 6.18, *df* = 1, *P* = 0.01) with control cages fledging all nestlings, whereas treatment cages only fledged 64% (±15.92) of the nestlings that hatched (Table S1).

**Figure 5:**
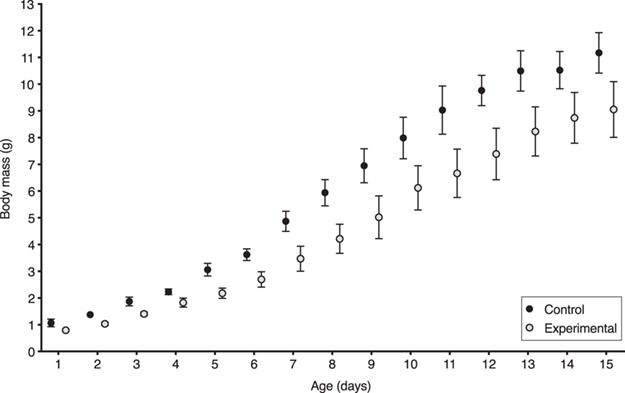
Nestling body mass gain from day hatched until fledging for the F2 generation of zebra finches comparing control versus permethrin-treated groups. Data for body mass are presented as a mean per nest. Error bars correspond to standard errors.

When we compared the effect of permethrin treatment by age on nestling growth we found that nestlings gained mass as they grew older (Fig. 5; *χ^2^* = 2537.39, *df* = 1, *P* < 0.0001). Overall, treatment had a marginally non-significant effect on nestling growth (*χ^2^* = 3.16, *df* = 1, *P* = 0.08), but the interaction between treatment and age significantly affected nestling growth (*χ^2^* = 13.04, *df* = 1, *P* = 0.0003), indicating that the effects of the permethrin treatment depended on the age of the nestlings.

Among the permethrin-treated group there were 2 cages whose progeny appeared underdeveloped at 14 days of age and most nestlings died. Cage 22 hatched 2 chicks out of 4 eggs laid and none survived. One of the nestlings died on day 4 and another nestling started to open its eyes at 14 days old. No feathers had emerged on the body of this nestling and all the primary feathers were still in sheaths 1–2 mm long (Fig. S1A, B, C). It weighed only 4.0 g at age 15 days and died at 20 days of age. The second such cage (cage 29) contained 7 eggs from which 3 nestlings hatched. One of these nestlings weighed 9.9 g at age 15 days, which is not abnormal. However, its moulting pattern was irregular. At 14 days of age, the primary feathers were still in sheaths 4 mm long with dorsal or head feather tracts starting to grow (Fig. S1D, E). At age 41 days, the nestling had not yet completed its initial moult with patches of bare skin visible (Fig. S1F, G). By age 62 days, its adult moult was not completed, with bare patches of skin exposed, and the primary feathers of the left wing still in sheaths (Fig. S1H). Another nestling died at age 7 days and a third at age 3 days. In contrast, the mean body mass for nestlings in the control group was 10.1 ± 0.5 g at 14 days old, their heads and bodies were covered in feathers, both ventrally and dorsally, and the primaries were fully unsheathed.

The raw data for the reproductive variables measured in this study for the F1.1, the F1.2 and the F2 generations are presented in Table S2.

### Permethrin detection in egg contents

Permethrin was detected inside some of the non-embryonated zebra finch eggs analysed. The concentration values obtained for each egg sample analysed are presented in Table S3. No permethrin was detected in any of the control eggs. Of the 21 eggs exposed to permethrin in the nest, 13 did not present detectable levels, 2 had trace amounts and 6 samples had measurable amounts of permethrin. Therefore, 28.5% (6 out of 21 eggs) exhibited levels within the LOQ. The most contaminated egg contained 4781 ng of permethrin per gram of dry egg mass, with an average of 1566 ng/g dry egg mass across the positive detections.

### Liver function tests

#### Parental generation

An *a priori* power analysis for sample size estimation for the repeated measures ANOVA suggested that our sample size was adequate to distinguish differences between treatment groups at *α* = 0.05 and power = 0.74. The effect size of 0.25 is considered to be medium using Cohen's (1988) criteria. The partial η^2^ suggests that the strength of relationship between variables was low. The data did not violate the equality of error (Levene’s Test; sig. >0.05) or equality of covariance assumptions (Box’s Test sig. >0.001) for any liver function test (AST, CK or BA).

Tests for the effect of permethrin-treatment were non-significant between the controls and permethrin-exposed birds for AST (*F*_(1,5)_ = 0.054, *P* = 0.83), BA (*F*_(1,5)_ = 3.28, *P* = 0.13) and CK (*F*_(1,5)_ = 0.074, *P* = 0.80). Tests for interaction effects between time and permethrin treatment were non-significant for AST (Wilk’s λ = 0.739, *F*_(2, 4)_ = 0.70, *P* = 0.546), BA (Wilk’s λ = 0.540, *F*_(2, 4)_ = 1.70, *P* = 0.292) and CK (Wilk’s λ = 0.58, *F*_(2, 4)_ = 1.460, *P* = 0.334), demonstrating that the levels of AST, BA and CK did not change significantly before permethrin exposure and at the time when the birds were exposed to permethrin. Tests for interaction effects between time and sex were also non-significant for two biomarkers, BA (Wilk’s λ = 0.63, *F*_(2, 4)_ = 1.17, *P* = 0.398) and CK (Wilk’s λ = 0.48, *F*_(2,4)_ = 2.19, *P* = 0.228). There was a significant interaction between time of testing and sex for AST (Wilk’s λ = 0.170, *F*_(2,4)_ = 9.75, *P* = 0.029); post-exposure AST levels for male birds were not significantly different from post-exposure AST levels for female birds (*F*_(1,5)_ = 0.755, *P* = 0.425). However, the initial AST measurements for male birds were higher on average than initial measurements for female birds. As a result, the overall main effect of time was significant for AST levels (Wilk’s λ = 0.17, *F*_(2,4)_ = 9.99, *P* = 0.028, partial η^2^ = 0.833), driven primarily by the interaction between sample time and sex of bird. In conclusion, none of three blood plasma biomarkers differed for the main effect of treatment; there was no significant difference between permethrin-treated or control groups in the parental generation.

#### F1 generation

There was no statistical difference between the F1.1 and F1.2 generations when comparing the combined three blood biomarkers AST, BA and CK at 6 weeks of age (*F*_(3,8)_ = 0.851, *P* = 0.504; Pillai’s trace = 0.990; partial η^2^ = 0.242). Neither were there any statistical differences between sexes (*F*_(3,8)_ = 0.427 *P* = 0.739; Pillai’s trace = 0.138; partial η^2^ = 0.138) nor between treatment groups (*F*_(3,8)_ = 0.283, *P* = 0.837; Pillai’s trace = 0.096; partial η^2^ = 0.096).

Within the F1.1 generation, there was no significant difference between experimental groups (*F*_(1,16)_ = 1.012, *P* = 0.329) or between baseline levels and post-exposure levels of AST (*F*_(1,16)_ = 0.265, *P* = 0.614), nor were there any differences in AST levels between sexes (*F*_(1,16)_ = 0.627, *P* = 0.440).

The main effect comparison of BA levels between sample times was significant (*F*_(1,16)_ = 7.534, *P* = 0.014); both permethrin treatment and control groups showed an increase in BA concentration over time. However, there was no significant difference in BA levels between treatment groups (*F*_(1,16)_ = 0.042, *P* = 0.84), nor were there any differences between sexes (*F*_(1,16)_ = 0.261, *P* = 0.616).

Finally, there was no significant difference between experimental groups (*F*_(1,13)_ = 0.364, *P* = 0.557) or between baseline levels and post-exposure levels of CK (*F*_(1,13)_ = 0.570, *P* = 0.464). There were also no differences in CK levels between sexes (*F*_(1,13)_ = 2.268, *P* = 0.156).

## Discussion

We found that permethrin had no significant effect on the number of eggs laid, the number of days from clutch initiation to hatching, the proportion of eggs that hatched, nestling sex ratio or fledgling body mass in either the F1 or F2 generations. However, finches in the permethrin group produced significantly larger broods than finches in the control group for the F1 generation, with the first clutch having a higher proportion of eggs hatch than the second clutch. Nestlings exposed to permethrin had lower body mass at hatching than control nestlings for both the F1 and F2 generations. These differences were statistically significant only for the F2 generation. Permethrin treatment had no effect on fledging success for the F1 generation, but clutch number significantly affected the number of fledglings produced in both control and permethrin treatments, with clutch 1 producing more fledglings than clutch 2. For the F2 generation, finches in the permethrin treatment exhibited lower fledging success than finches in the control group. Birds in the F1 generation were able to reproduce and fledge offspring after continued exposure to permethrin as eggs, nestlings and after sitting on treated materials themselves. Thus, permethrin does not cause infertility in zebra finches within this timeframe.

While risk assessments based on studies on adult chicken and waterfowl suggest that permethrin has low toxicity for birds (Mineau *et al*., 2001; Meléndez *et al*., 2006), other studies argue against the generalizability of these conclusions as domestic birds are less sensitive to toxicants, nestlings are often more sensitive than adults and altricial nestlings are more sensitive than precocial nestlings (reviewed in Hund *et al*., 2015). Our observations of some hatchlings with significantly reduced body mass, slower growth rates and repressed feather growth in the permethrin treatment group point towards sub-lethal effects of this insecticide. However, because we sought to simulate the technique being used in the field for ectoparasite management and did not quantify the extent of permethrin exposure per finch pair or per nestling, there could have been some degree of variability in exposure. This variability might be the reason why some nestlings showed adverse effects while others did not. Our findings are similar to those in a study where a mix of permethrin, tetramethrin and piperonyl butoxide was applied to nests and nest boxes of wild pied flycatchers (López-Arrabé *et al*., 2014).

Although not significantly different, egg production was higher in nests treated with permethrin, and consequently, permethrin-treated nests had significantly larger broods than control nests for the F1 generation, which is suggestive of a stimulatory effect of permethrin. Such a stimulatory effect could be metabolic, a behavioural response (i.e. parents feeding more), or might be mediated by hormones as seen in other taxa. To the best of our knowledge, nothing is known about how permethrin and its metabolites affect the avian endocrine system. In fish, permethrin and its metabolites have endocrine activity *in vivo*. These can induce estrogen-dependent egg proteins in male fish (Nillos *et al*., 2010). These compounds act as an estrogen receptor agonist *in vivo* in fish, while in cultured mammalian cells, the unmetabolized compound acts as an estrogen receptor antagonist (Brander *et al*., 2012). In humans, permethrin metabolites are capable of interacting with the human estrogen receptor as they mimic estrogen (McCarthy *et al*., 2006). Our results showing a non-statistically significant increase in egg production in finches exposed to permethrin are worth following up with larger sample sizes and for more generations.

Hatchlings in the permethrin-treated group had lower body mass than hatchlings in the control group for both the F1 and F2 generations although the differences were statistically significant only for the F2 generation. Food in the experiment was provided *ad libitum* and although we did not record begging intensity or food provisioning by the parents, the fact that control and permethrin-treated nestlings did not differ in body mass at fledging suggests that parents that were exposed to permethrin might have provided more food to hatchlings to compensate for their lower body mass at hatch. Rapid compensatory body mass growth has been previously described in zebra finch nestlings in a food restriction experiment (Killpack *et al*., 2014).

We do not know which metabolic pathways were disrupted by permethrin or its metabolites that resulted in the observed repressed feather growth in some permethrin-exposed nestlings. *In vitro* cell culture experiments have shown that permethrin can decrease neural crest cell migration (Nyffeler *et al*., 2017). Adequate neural crest cell migration is required for the normal spatiotemporal development of some feather tracts, especially those in the craniofacial region (Eames and Schneider, 2005; Schneider, 2018). Reductions in neural crest cell migration could produce some of the observed feather abnormalities, although genes related to feather development or branching (Yu *et al*., 2002; Darnell *et al*., 2014; Lowe *et al*., 2014) were likely involved as well.

We found no detrimental effects of permethrin treatment on liver function tests for any generation. This result is important given that the liver is the main detoxification organ, and it produces many proteins of importance in egg-laying species (see below). The two main liver function test enzymes are AST and alanine aminotransferase (ALT), both of which are related to liver damage and changes in hepatic function (Manna *et al*., 2004). In birds, causes for increased levels of ALT are cell damage in general, so this non-specific enzyme has limited usefulness. Further, ALT increases with hemolysis, making high values difficult to interpret (Hochleithner, 1994); because of this we did not test levels of ALT in our study. Increases of serum activity of AST are also indicative of muscle damage (Manna *et al*., 2004). Increased CK activities are mostly due to muscle cell damage; therefore, we assayed CK to help distinguish muscle from liver cell damage (Hochleithner, 1994). By assaying CK levels, muscle damage can be excluded as the cause for elevated AST values. Bile acids are sensitive indicators of liver function. Elevation of BAs is consistent with decreased liver function (Lumeij, 1999). Thus, BA assays are useful in determining chronic, long-standing and non-inflammatory states of decreased hepatic function that may not be reflected in hepatic enzyme elevation (Hochleithner *et al*., 2005). The fact that BA values increased significantly for the F1.1 generation between the time when birds were 6 weeks old and later during egg incubation is likely due to normal development, as the increase was recorded for both the permethrin-treated and the control birds. Bile acids are known to increase over the first 6 weeks of development in domestic fowl (*Gallus gallus domesticus*; Green and Kellog, 1987), so it is reasonable to conclude that the significant main effect in our analysis reflects a maturation of either the digestive system or of serum lipid transport over time. Barn owlets whose nests were treated with a microencapsulated formulation of permethrin showed no differences in any blood biomarker measured, including AST and CK, when compared to owlets in untreated or control nests (Efstathion, 2015). No biochemical changes were found in rats when orally administered low doses of permethrin (24–60 mg kg^−1^ body weight per day) but at higher doses (80–120 mg kg^−1^ body weight per day) AST values increased (Shah and Gupta, 1997).

We detected measurable levels of permethrin inside 28.5% of the zebra finch eggs analyzed. Residues of permethrin have been reported in eggs of a range of bird species in the wild as well as in poultry (Causton and Lincango, 2014; Corcellas *et al*., 2017), though it is not clear what the effects of this contamination are. In our experiment, eggs were subject to direct contact with permethrin through adult finches sitting on and the eggs resting on permethrin-treated materials, which may have allowed the permethrin to permeate the eggshell and accumulate inside the eggs. Secondary contamination may also have been possible via parents holding permethrin-treated nesting materials in their beaks or by maternal transfer to developing ova through inhalation exposure (Bro *et al*., 2016). Lipovitellin, phosvitin and some yolk lipoproteins are synthesized by the liver of the laying female (Sauveur and de Reviers, 1988), and it is feasible that the fatty egg content could incorporate permethrin, a lipophilic substance. Future research designed to disentangle the effects of maternal transfer versus direct contamination of the eggs is encouraged.

Taken together, our findings of smaller hatchling body mass for permethrin-exposed nestlings, the significant interaction between permethrin treatment and age for body mass of the hatchlings, the lower fledging success of the offspring of permethrin-treated birds for the F2 generation, the cases of underdeveloped nestlings or incomplete moulting described and the fact that permethrin was detected inside some eggs suggest sub-lethal effects overall when finches are exposed continuously to permethrin in nests. Nevertheless, these results could also be partially confounded by inbreeding, and other in-cage effects. The high proportion of unhatched eggs in our study, in both the permethrin-treated and control groups, points towards inbreeding; genetic similarity between parents is known to reduce hatching success in birds (Bensch *et al*., 1994). Moreover, inbreeding is a known cause of fitness reduction in zebra finches (reviewed by Ihle and Forstmeier, 2013). Across bird species, an estimated hatching failure of 15% is common (reviewed by Ihle *et al*., 2013). For wild zebra finches, hatching failure is not uncommon; ~16% of the eggs fail to hatch (Zann, 1996; Griffith *et al*., 2008). For some captive populations, even higher rates of hatching failure have been reported (e.g. 35%; Forstmeier and Ellegren, 2010), similar to the hatching failure rates observed in our study. A larger sample size for the F2 generation would be needed to make a definitive conclusion.

It is important for conservation management purposes to appreciate that our study represents a worse-case scenario of exposure of breeding passerines to permethrin compared to the study of Knutie *et al*. (2014) where finches self-fumigated nests or the techniques currently being evaluated for parasite management in the wild. In our study, adult birds, eggs, nestlings and subsequently their offspring were continually exposed to nest material that had been treated in its entirety with 1% emulsifiable permethrin spray. Using the same product that we used in our trial, Knutie *et al*. (2014) in their field study estimated that an average of 4.3% of the nesting materials incorporated by the Galapagos finches into their nests was cotton that had been treated with 1% permethrin. This amount was sufficient to reduce *P. downsi* larval numbers and increase reproductive success of finches.

Field trials currently underway in the Galapagos Islands are testing methods that lower the risks of direct exposure to permethrin. The first application of permethrin is carried out mid to late incubation (rather than prior to incubation), and permethrin is injected into the base of the nest (not on the nest surface where eggs and hatchlings sit). Unlike our study, the trials in Galapagos are testing the application of 0.5% of a microencapsulated, slow-release form of permethrin (Fessl *et al*., 2018; Causton *et al*., 2019). Depending on the size and type of nest, 1 or 2 doses of 1–5 ml of slow-release permethrin is delivered to the nest. However, this technique can only be used for nests that can be reached. For nests that are out of reach and for threatened bird species with patchy distributions over large areas, the self-fumigation technique offers a solution in the short term.

In conclusion, our study indicates that continued exposure to 1% permethrin can have sub-lethal effects on growth and reproductive success, but not on liver function or fertility, of small passerines. Therefore, for conservation purposes, permethrin should only be used to treat wild bird nests against ectoparasites in the field if its benefits to bird body condition and reproductive success are greater than when no action is taken. Additional studies are needed to evaluate formulations and doses of permethrin used in field conditions, in particular permethrin persistence in the nest material, extent of dermal exposure and potential adverse effects of intergenerational exposure. Until a permanent solution is found to combat the threat of *P. downsi* to the endemic birds of the Galapagos Islands, the increased nestling survival due to the judicious application of permethrin in nests seems to outweigh the potential sub-lethal costs to nestlings and might prevent the imminent extinction of some of the rarest species of Darwin’s finches.

## Supplementary Material

Suppl-Fig1_coaa076

Suppl-Info-revision2_coaa076
