# Supplementary figures and images for: Sub-lethal effects of permethrin exposure on a passerine: implications for managing ectoparasites in wild bird nests
Source: Conserv Physiol. 2020 Sep 8;8(1):coaa076. doi: 10.1093/conphys/coaa076 (PMC7416766; doi:10.1093/conphys/coaa076)

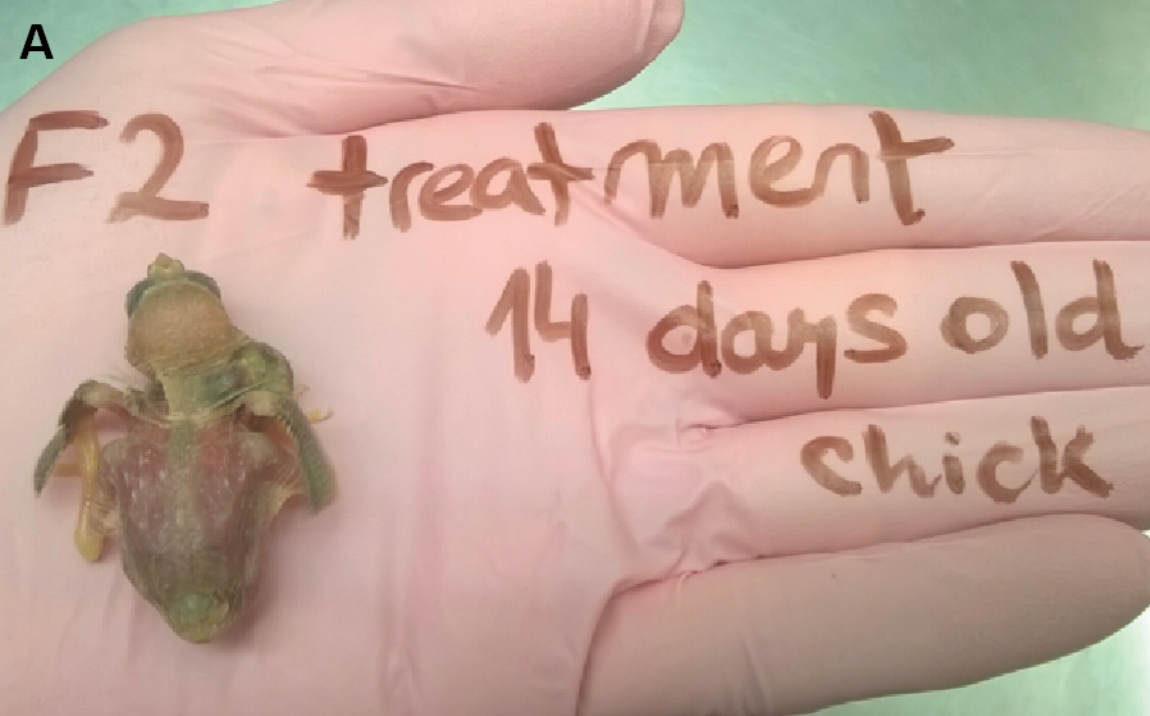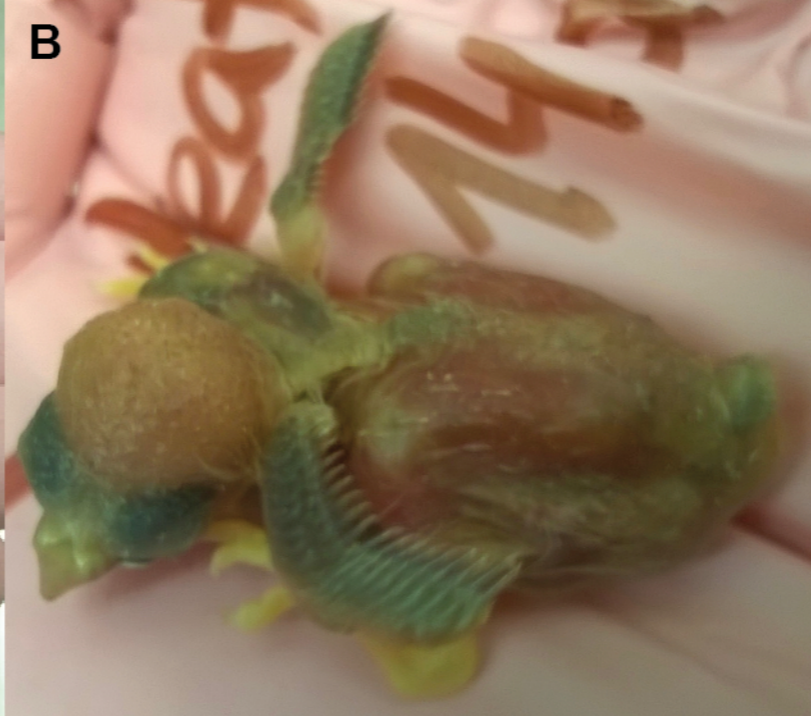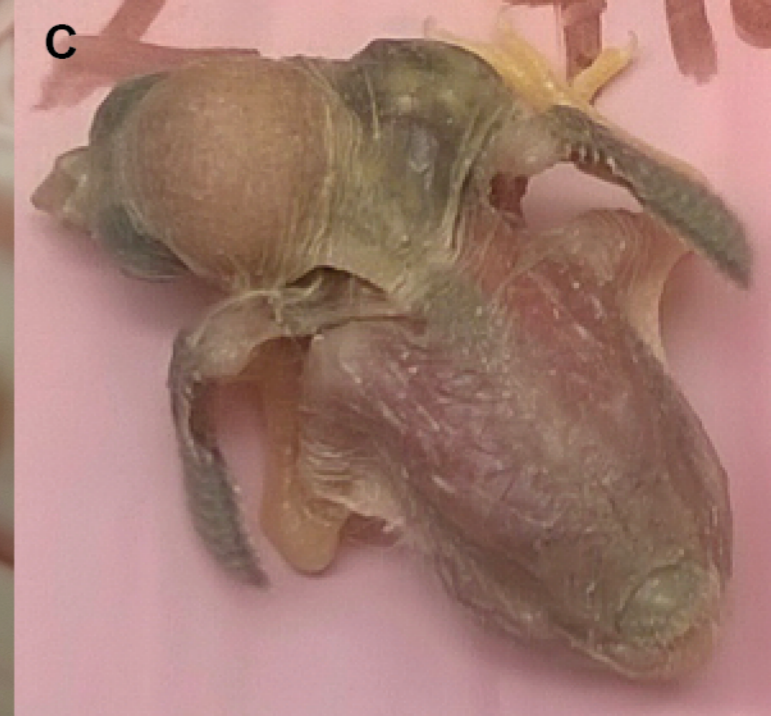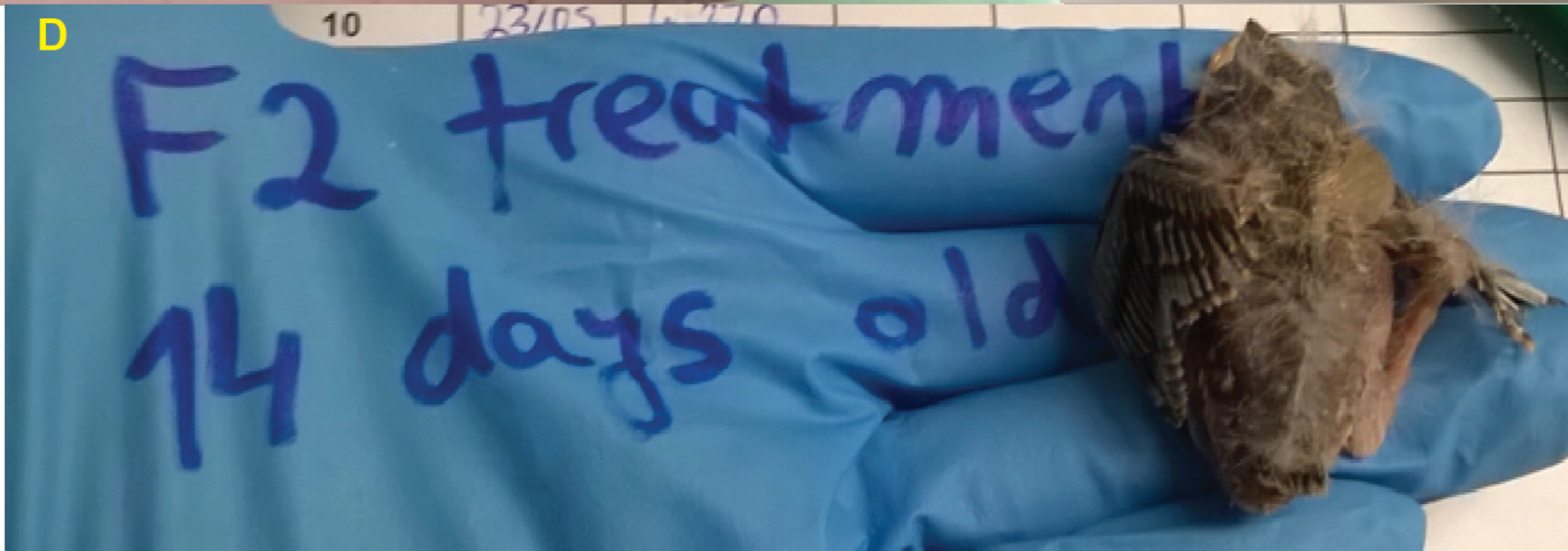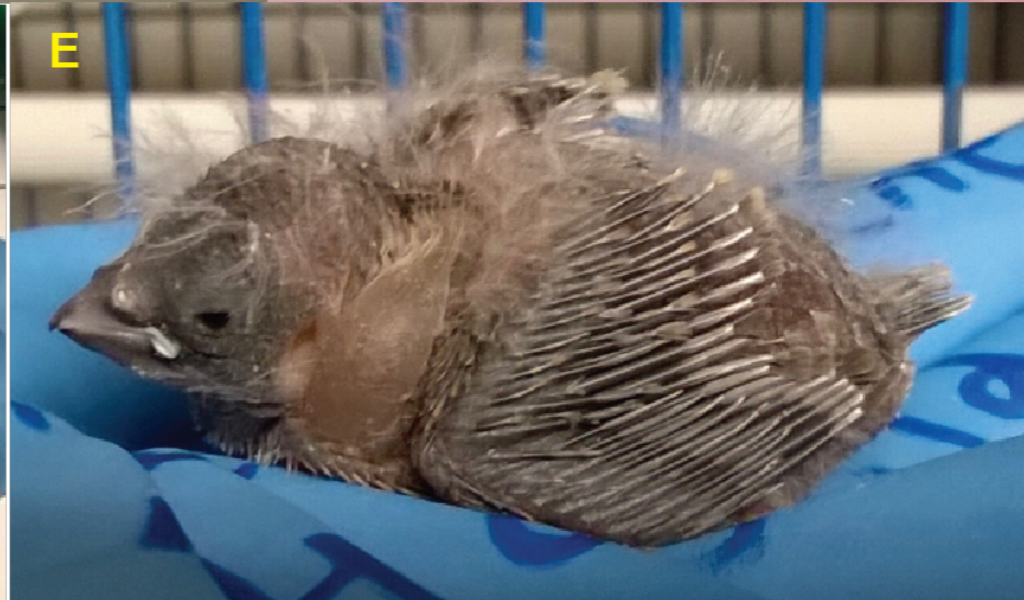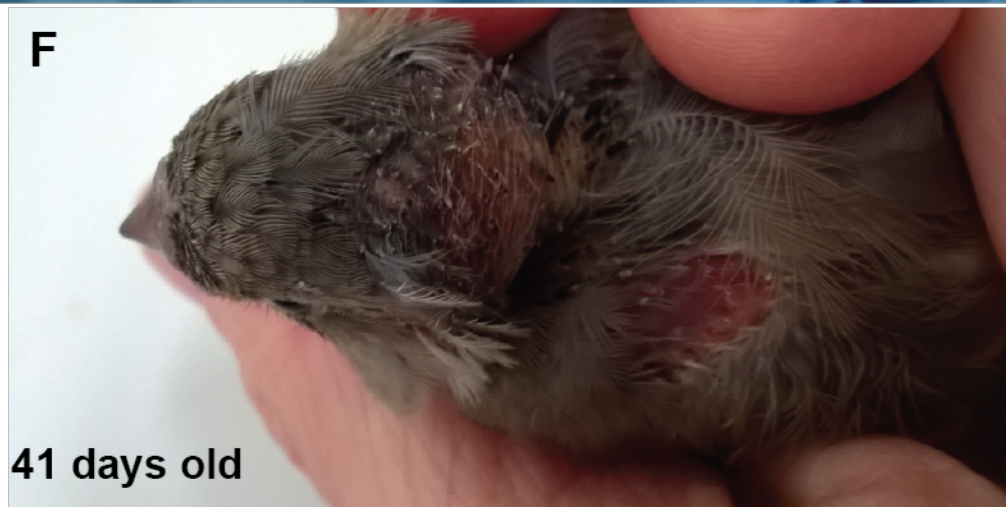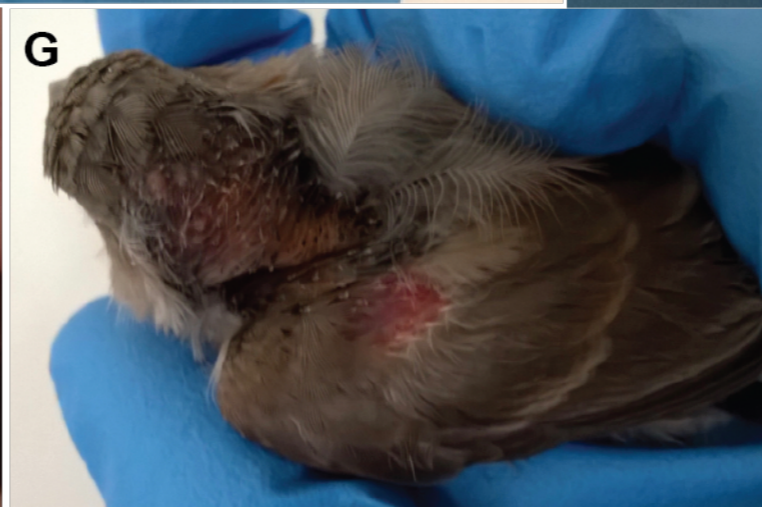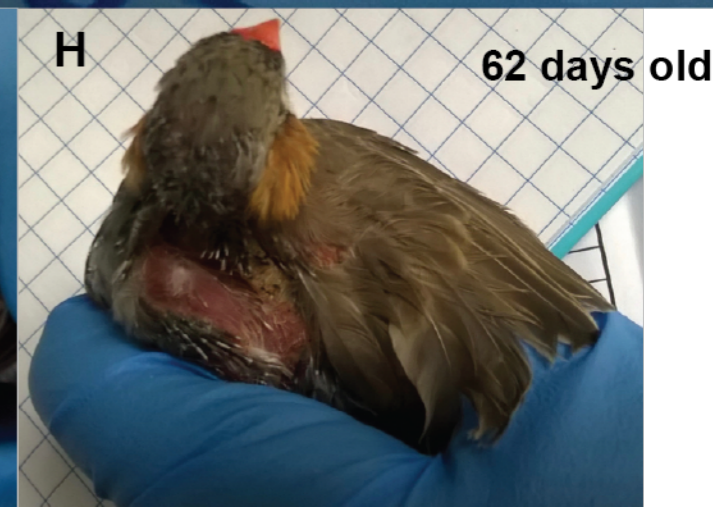

Supplement: Suppl-Fig1_coaa076 [file suppl-fig1_coaa076.pdf]
