# Supplementary material for: Sub-lethal effects of permethrin exposure on a passerine: implications for managing ectoparasites in wild bird nests
Source: Conserv Physiol. 2020 Sep 8;8(1):coaa076. doi: 10.1093/conphys/coaa076 (PMC7416766; doi:10.1093/conphys/coaa076)
Supplement: Suppl-Info-revision2_coaa076 [file suppl-info-revision2_coaa076.docx]

**Sub-lethal effects of permethrin exposure on a passerine: Implications for managing ectoparasites in wild bird nests**

Mariana Bulgarella, Sarah A. Knutie, Margaret A. Voss, Francesca Cunninghame, Brittany J. Florence-Bennett, Gemma Robson, Robert A. Keyzers, Lauren M. Taylor, Philip J. Lester, George E. Heimpel and Charlotte E. Causton

**Supplemental Figure 1.** Underdeveloped nestlings hatched from the second generation of zebra finches (*Taeniopygia guttata*) exposed to permethrin during egg formation, incubation, while in nest as a hatchling and later while building their own nests and sitting on eggs on treated materials. (A, B, C) Fourteen day old nestling from permethrin-treated cage 22, showing the lack of feathers on the body and head and primary feathers still in sheaths 1–2 mm long. (D, E) Fourteen day old nestling from permethrin-treated cage 29, primary feathers were still in sheaths 4 mm long with dorsal or head feather tracts starting to grow. (F, G) Same nestling from cage 29 at age 41 days, incomplete initial moult with patches of bare skin visible on head and left shoulder. (H) Same nestling from cage 29 at age 62 days, adult moult not completed yet, with bare patches of skin exposed, and the primary feathers of the left wing still in sheaths.

**
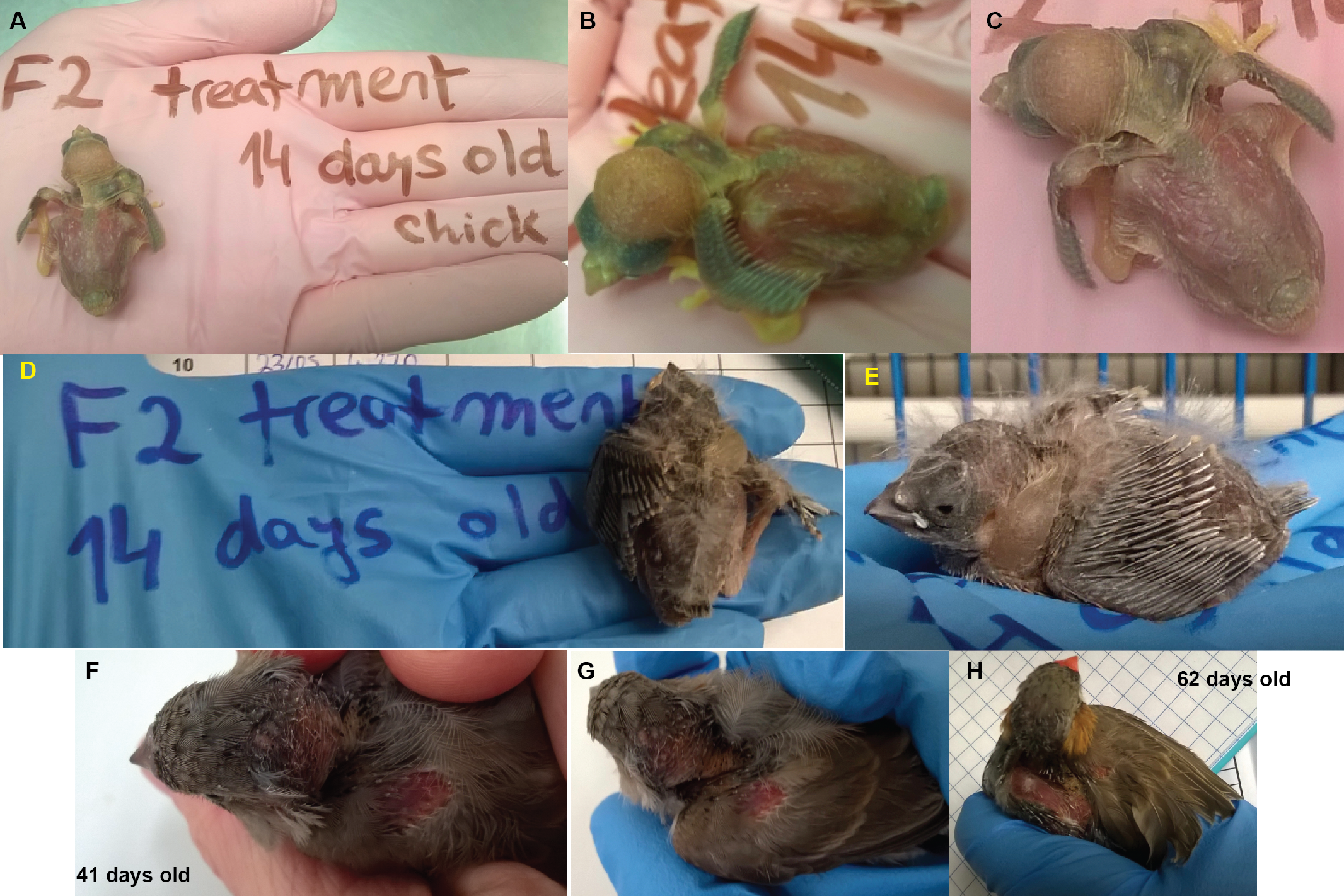
**

**Table S1.** Comparison of the average clutch size, egg hatch rates and nestling survival rates by treatment group and by clutch number for the F1 and F2 generations of zebra finches. Values presented are means ± standard errors.

| **Generation and experimental group (*n*)** | **Clutch** | **Average clutch size** | **Mean egg hatching rates (%)** | **Mean nestling survival rates (%)** |
| --- | --- | --- | --- | --- |
| F1.1 permethrin treatment (10) | 1^st^ | 5.00 ± 0.46 | 73.46 ± 6.46 | 79.66 ± 6.47 |
| F1.1 control (10) | 1^st^ | 4.20 ± 0.34 | 55.02 ± 8.57 | 95.00 ± 4.74 |
| F1.2 permethrin treatment (9) | 2^nd^ | 5.22 ± 0.76 | 44.14 ± 5.11 | 68.52 ± 13.47 |
| F1.2 control (9) | 2^nd^ | 3.55 ± 0.38 | 50.74 ± 4.88 | 100.00 ± 0.00 |
| F2 permethrin treatment (6) | 1^st^ | 4.50 ± 0.56 | 67.70 ± 10.99 | 63.89 ± 15.92 |
| F2 control (4) | 1^st^ | 3.25 ± 0.21 | 75.00 ± 13.82 | 100.00 ± 0.00 |

**Table S2.** Reproductive variables measured for each parental pair of zebra finch (*Taeniopygia guttata*) in the experiment (*n* = 20) and for the F1.1 generation (*n* = 10).

| **Cage** | **Clutch number** | **Date 1st egg was laid** | **Date 1st chick hatched** | **Incubation days** | **Total # eggs laid** | **# eggs that hatched** | **# chicks that survived AND fledged** | **Nestling sex** | **Band colour and number** | **Nestling body mass at 42 days old (g)** | **Group** |
| --- | --- | --- | --- | --- | --- | --- | --- | --- | --- | --- | --- |
| **1** | 1st | 14-Mar-17 | 13-Apr-17 | 30 | 8 | 5 | 5 | M | Red 3 |  | treatment |
|  |  |  |  |  |  |  |  | F | Red 4 |  |  |
|  |  |  |  |  |  |  |  | F | Red 5 |  |  |
|  |  |  |  |  |  |  |  | M | Red 6 |  |  |
|  |  |  |  |  |  |  |  | F | Red 7 |  |  |
|  | 2nd | 31-May-17 | 27-Jun-17 | 27 | 9 | 2 | 2 | ? | Not banded | 14.395 |  |
|  |  |  |  |  |  |  |  | ? | Not banded | 14.450 |  |
|  |  |  |  |  |  |  |  |  |  |  |  |
| **2** | 1st | 22-Jun-18 | 8-Jul-18 | 16 | 5 | 5 | 4 | F | Lime green 6 | 15.485 | treatment |
|  |  |  |  |  |  |  |  | F | Lime green 7 | 14.620 |  |
|  |  |  |  |  |  |  |  | M | Lime green 8 | 14.045 |  |
|  |  |  |  |  |  |  |  | M | Lime green 9 | 14.560 |  |
|  | 2nd | 8-Sep-18 | 27-Sep-18 | 19 | 6 | 2 | 2 | F | Not banded | 13.130 |  |
|  |  |  |  |  |  |  |  | M | Not banded | 15.370 |  |
|  |  |  |  |  |  |  |  |  |  |  |  |
| **3** | 1st | 27-May-18 | 12-Jun-18 | 16 | 6 | 3 | 2 | F | Dark blue 4 | 14.470 | treatment |
|  |  |  |  |  |  |  |  | F | Dark blue 5 | 14.375 |  |
|  | 2nd | 15-Jul-18 | 3-Aug-18 | 19 | 5 | 3 | 3 | M | Not banded | 13.920 |  |
|  |  |  |  |  |  |  |  | M | Not banded | 12.295 |  |
|  |  |  |  |  |  |  |  | F | Not banded | 12.625 |  |
|  |  |  |  |  |  |  |  |  |  |  |  |
| **4** | 1st | 28-Aug-17 | 24-Sep-17 | 27 | 4 | 3 | 1 | M | Orange 3 | 8.400 | treatment |
|  | 2nd | 25-Oct-17 | 13-Nov-17 | 19 | 3 | 1 | 0 |  | Dead |  |  |
|  |  |  |  |  |  |  |  |  |  |  |  |
| **5** | 1st | 13-Dec-17 | 31-Dec-17 | 18 | 7 | 4 | 3 | F | Dark Blue 1 | 15.065 | treatment |
|  |  |  |  |  |  |  |  | M | Light Blue 2 | 14.890 |  |
|  |  |  |  |  |  |  |  | M | Light Blue 3 | 15.310 |  |
|  | 2nd | 16-Apr-18 | 4-May-18 | 18 | 4 | 2 | 1 | M | Not banded | 13.725 |  |
|  |  |  |  |  |  |  |  |  |  |  |  |
| **6** | 1st | 19-Aug-18 | 7-Sep-18 | 19 | 3 | 3 | 3 | M | Not banded | 13.165 | treatment |
|  |  |  |  |  |  |  |  | M | Not banded | 13.220 |  |
|  |  |  |  |  |  |  |  | F | Not banded | 13.340 |  |
|  |  |  |  |  |  |  |  |  |  |  |  |
| **7** | 1st | 3-Aug-18 | 18-Aug-18 | 15 | 4 | 3 | 3 | M | Lime green 10 | 14.990 | treatment |
|  |  |  |  |  |  |  |  | F | Not banded | 16.470 |  |
|  |  |  |  |  |  |  |  | F | Not banded | 14.215 |  |
|  | 2nd | 20-Sep-18 | 7-Oct-18 | 17 | 5 | 2 | 2 | M | Not banded |  |  |
|  |  |  |  |  |  |  |  | F | Not banded |  |  |
|  |  |  |  |  |  |  |  |  |  |  |  |
| **8** | 1st | 4-Sep-17 | 21-Sep-17 | 17 | 4 | 3 | 2 | F | Orange 5 | 13.480 | treatment |
|  |  |  |  |  |  |  |  | M | Orange 6 | 15.060 |  |
|  | 2nd | 26-Oct-17 | 15-Nov-17 | 20 | 4 | 3 | 2 | F | Not banded | 12.795 |  |
|  |  |  |  |  |  |  |  | M | Not banded | 14.165 |  |
|  |  |  |  |  |  |  |  |  |  |  |  |
| **9** | 1st | 23-Aug-17 | 10-Sep-17 | 18 | 5 | 2 | 2 | F | Orange 1 | 11.895 | treatment |
|  |  |  |  |  |  |  |  | F | Orange 2 | 12.005 |  |
|  | 2nd | 8-Oct-17 | 31-Oct-17 | 23 | 9 | 3 | 0 |  | Dead |  |  |
|  |  |  |  |  |  |  |  |  |  |  |  |
| **10** | 1st | 5-Mar-18 | 20-Mar-18 | 15 | 4 | 4 | 3 | F | Not banded | 13.785 | treatment |
|  |  |  |  |  |  |  |  | F | Not banded | 12.315 |  |
|  |  |  |  |  |  |  |  | F | Not banded | 13.480 |  |
|  | 2nd | 29-Sep-18 | 14-Oct-18 | 15 | 2 | 1 | 1 | M | Not banded |  |  |
|  |  |  |  |  |  |  |  |  |  |  |  |
| **11** | 1st | 10-Oct-17 | 26-Oct-17 | 16 | 4 | 4 | 4 | F | White 8 | 14.400 | control |
|  |  |  |  |  |  |  |  | M | White 9 | 13.970 |  |
|  |  |  |  |  |  |  |  | M | White 7 | 13.145 |  |
|  |  |  |  |  |  |  |  | M | White 10 | 13.110 |  |
|  | 2nd | 17-Jan-18 | 1-Feb-18 | 15 | 3 | 2 | 2 | M | Not banded | 14.600 |  |
|  |  |  |  |  |  |  |  | F | Not banded | 14.185 |  |
|  |  |  |  |  |  |  |  |  |  |  |  |
| **12** | 1st | 17-Oct-17 | 2-Nov-17 | 16 | 4 | 2 | 1 | M | Lime green 1 | 13.190 | control |
|  | 2nd | 4-Jul-18 | 22-Jul-18 | 18 | 5 | 1 | 1 | F | Not banded |  |  |
|  |  |  |  |  |  |  |  |  |  |  |  |
| **13** | 1st | 7-Sep-17 | 28-Sep-17 | 21 | 3 | 1 | 1 | F | White 1 |  | control |
|  | 2nd | 31-Oct-17 | 19-Nov-17 | 19 | 2 | 1 | 1 | F | Not banded | 13.145 |  |
|  |  |  |  |  |  |  |  |  |  |  |  |
| **14** | 1st | 26-Feb-18 | 15-Mar-18 | 17 | 3 | 1 | 1 | M | Dark blue 2 | 14.165 | control |
|  | 2nd | 20-May-18 | 6-Jun-18 | 17 | 3 | 1 | 1 | M | Not banded | 14.185 |  |
|  |  |  |  |  |  |  |  |  |  |  |  |
| **15** | 1st | 5-Sep-17 | 24-Sep-17 | 19 | 4 | 4 | 4 | M | White 6 |  | control |
|  |  |  |  |  |  |  |  | M | White 3 |  |  |
|  |  |  |  |  |  |  |  | F | White 5 |  |  |
|  |  |  |  |  |  |  |  | M | White 4 |  |  |
|  | 2nd | 31-Jan-18 | 21-Feb-18 | 21 | 4 | 2 | 2 | F | Not banded | 12.915 |  |
|  |  |  |  |  |  |  |  | F | Not banded | 12.860 |  |
|  |  |  |  |  |  |  |  |  |  |  |  |
| **16** | 1st | 23-Feb-17 | 13-Mar-17 | 18 | 4 | 2 | 2 | M | Yellow 1 | 13.055 | control |
|  |  |  |  |  |  |  |  | F | Yellow 2 | 14.090 |  |
|  | 2nd | 10-Apr-17 | 27-Apr-17 | 17 | 5 | 3 | 3 | F | Not banded |  |  |
|  |  |  |  |  |  |  |  | F | Not banded |  |  |
|  |  |  |  |  |  |  |  | M | Not banded |  |  |
|  |  |  |  |  |  |  |  |  |  |  |  |
| **17** | 1st | 27-May-17 | 21-Jun-17 | 25 | 5 | 4 | 4 | F | Not banded |  | control |
|  |  |  |  |  |  |  |  | F | Not banded |  |  |
|  |  |  |  |  |  |  |  | F | Not banded |  |  |
|  |  |  |  |  |  |  |  | M | Not banded |  |  |
|  |  |  |  |  |  |  |  |  |  |  |  |
| **18** | 1st | 19-Feb-18 | 10-Mar-18 | 19 | 7 | 2 | 2 | M | Not banded | 13.075 | control |
|  |  |  |  |  |  |  |  | F | Dark Blue 3 | 13.415 |  |
|  | 2nd | 8-Apr-18 | 27-Apr-18 | 19 | 5 | 3 | 3 | M | Not banded | 14.850 |  |
|  |  |  |  |  |  |  |  | M | Not banded | 11.525 |  |
|  |  |  |  |  |  |  |  | F | Not banded | 13.955 |  |
|  |  |  |  |  |  |  |  |  |  |  |  |
| **19** | 1st | 26-Feb-17 | 13-Mar-17 | 15 | 4 | 1 | 1 | M | Yellow 7 | 12.015 | control |
|  | 2nd | 20-Dec-17 | 9-Jan-18 | 20 | 2 | 1 | 1 | F | Not banded | 12.760 |  |
|  |  |  |  |  |  |  |  |  |  |  |  |
| **20** | 1st | 26-Feb-18 | 15-Mar-18 | 17 | 4 | 2 | 2 | F | Lime green 3 | 15.755 | control |
|  |  |  |  |  |  |  |  | M | Not banded | 15.980 |  |
|  | 2nd | 10-Apr-18 | 5-May-18 | 25 | 3 | 2 | 2 | M | Not banded | 15.105 |  |
|  |  |  |  |  |  |  |  | M | Not banded | 16.115 |  |
|  |  |  |  |  |  |  |  |  |  |  |  |
| **F1.1 crosses producing the F2 generation** | | | | |  |  |  |  |  |  |  |
| **21** | 1st | 1-Jul-17 | 26-Jul-17 | 25 | 3 | 1 | 1 | M | Red 8 | 14.560 | control |
|  |  |  |  |  |  |  |  |  |  |  |  |
| **22** | 1st | 2-Jan-18 | 24-Jan-18 | 22 | 4 | 2 | 0 |  | Dead |  | treatment |
|  |  |  |  |  |  |  |  |  |  |  |  |
| **23** | 1st | 23-Aug-18 | 9-Sep-18 | 17 | 5 | 4 | 2 | F | Not banded | 12.740 | treatment |
|  |  |  |  |  |  |  |  | F | Not banded | 13.905 |  |
|  |  |  |  |  |  |  |  |  |  |  |  |
| **25** | 1st | 22-Mar-18 | 9-Apr-18 | 18 | 3 | 3 | 3 | F | Not banded | 12.260 | control |
|  |  |  |  |  |  |  |  | F | Not banded | 12.905 |  |
|  |  |  |  |  |  |  |  | M | Not banded | 13.325 |  |
|  |  |  |  |  |  |  |  |  |  |  |  |
| **27** | 1st | 24-Jun-18 | 8-Jul-18 | 14 | 3 | 1 | 1 | M | Dark blue 6 | 12.420 | treatment |
|  |  |  |  |  |  |  |  |  |  |  |  |
| **28** | 1st | 19-Oct-18 | 4-Nov-18 | 16 | 4 | 4 | 4 | F | Not banded |  | control |
|  |  |  |  |  |  |  |  | M | Not banded |  |  |
|  |  |  |  |  |  |  |  | F | Not banded |  |  |
|  |  |  |  |  |  |  |  | M | Not banded |  |  |
|  |  |  |  |  |  |  |  |  |  |  |  |
| **29** | 1st | 28-Apr-18 | 14-May-18 | 16 | 7 | 3 | 1 | M | Not banded | 15.535 | treatment |
|  |  |  |  |  |  |  |  |  |  |  |  |
| **30** | 1st | 24-Jul-18 | 10-Aug-18 | 17 | 3 | 2 | 2 | M | Not banded | 14.065 | control |
|  |  |  |  |  |  |  |  | M | Not banded | 14.140 |  |
|  |  |  |  |  |  |  |  |  |  |  |  |
| **32** | 1st | 19-Oct-18 | 4-Nov-18 | 16 | 3 | 3 | 3 | F | Not banded |  | treatment |
|  |  |  |  |  |  |  |  | F | Not banded |  |  |
|  |  |  |  |  |  |  |  | M | Not banded |  |  |
|  |  |  |  |  |  |  |  |  |  |  |  |
| **33** | 1st | 2-Oct-18 | 18-Oct-18 | 16 | 5 | 5 | 5 | F | Not banded |  | treatment |
|  |  |  |  |  |  |  |  | M | Not banded |  |  |
|  |  |  |  |  |  |  |  | M | Not banded |  |  |
|  |  |  |  |  |  |  |  | F | Not banded |  |  |
|  |  |  |  |  |  |  |  | F | Not banded |  |  |
|  |  |  |  |  |  |  |  |  |  |  |  |

**Table S3.** Concentration levels (expressed in ng per g of dry egg mass) of permethrin detected in control and treated eggs collected from zebra finches (*Taeniopygia guttata*) in this study. ND = not detected, TR = trace levels.

| Sample ID | Permethrin content (µg) | Sample type | Egg mass (g, wet) | Egg mass (g, dry) | % dry egg mass | Recovery  (%) |  | Amount (µg) per g dry egg | Amount (ng) per g dry egg |
| --- | --- | --- | --- | --- | --- | --- | --- | --- | --- |
| Egg 1 | 0.057 | Spiked egg | 0.523 | 0.104 | 19.9 | ND |  |  |  |
| Egg 2 | 0.056 | Spiked egg | 0.464 | 0.105 | 22.6 | 75.7 |  |  |  |
| Egg 3 | ND | Spiked egg | 0.754 | 0.164 | 21.7 | 74.9 |  |  |  |
| Egg 4 | 0.063 | Spiked egg | 0.755 | 0.169 | 22.4 | ND |  |  |  |
| Egg 5 | 0.065 | Spiked egg | 0.620 | 0.132 | 21.2 | 84.2 |  |  |  |
| Egg 6 | ND | Spiked egg | 0.699 | 0.165 | 23.7 | 86.5 |  |  |  |
| Egg 7 | ND | Spiked egg | 0.773 | 0.146 | 18.8 | 81.4 |  |  |  |
| Egg 8 | 0.073 | Spiked egg | 0.643 | 0.178 | 27.7 | ND |  |  |  |
| Egg 9 | 0.058 | Spiked egg | 0.557 | 0.137 | 24.7 | 97.5 |  |  |  |
| Egg 10 | 0.080 | Spiked egg | 0.480 | 0.136 | 28.4 | 77.1 |  |  |  |
| Egg 11 | 0.038 | Spiked egg | 0.639 | 0.160 | 25.0 | 107.0 |  |  |  |
| Egg 12 | 0.057 | Spiked egg | 0.787 | 0.153 | 19.4 | 51.0 |  |  |  |
| Egg 13 | 0.056 | Spiked egg | 0.578 | 0.119 | 20.6 | ND |  |  |  |
| Egg 14 | ND | Control egg | 0.524 | 0.139 | 26.5 |  |  |  |  |
| Egg 15 | ND | Control egg | 0.524 | 0.139 | 26.5 |  |  |  |  |
| Egg 16 | ND | Control egg | 0.533 | 0.138 | 25.8 |  |  |  |  |
| Egg 17 | ND | Control egg | 0.527 | 0.127 | 24.1 |  |  |  |  |
| Egg 18 | ND | Control egg | 0.527 | 0.121 | 22.8 |  |  |  |  |
| Egg 19 | ND | Control egg | 0.527 | 0.121 | 22.8 |  |  |  |  |
| Egg 20 | ND | Control egg | 0.459 | 0.116 | 25.3 |  |  |  |  |
| Egg 21 | ND | Control egg | 0.459 | 0.116 | 25.3 |  |  |  |  |
| Egg 22 | ND | Control egg | 0.459 | 0.123 | 26.8 |  |  |  |  |
| Egg 23 | ND | Control egg | 1.011 | 0.193 | 19.1 |  |  |  |  |
| Egg 24 | ND | Control egg | 1.134 | 0.203 | 17.9 |  |  |  |  |
| Egg 25 | ND | Control egg | 1.070 | 0.195 | 18.2 |  |  |  |  |
| Egg 26 | ND | Control egg | 0.947 | 0.173 | 18.3 |  |  |  |  |
| Egg 27 | ND | Control egg | 0.922 | 0.200 | 21.6 |  |  |  |  |
| Egg 28 | ND | Control egg | 0.989 | 0.214 | 21.6 |  |  |  |  |
| Egg 29 | ND | Treatment egg | 0.787 | 0.158 | 20.1 |  |  |  |  |
| Egg 30 | 0.127 | Treatment egg | 0.807 | 0.184 | 22.8 |  |  | 0.693 | 692.9 |
| Egg 31 | ND | Treatment egg | 0.990 | 0.204 | 20.6 |  |  |  |  |
| Egg 32 | ND | Treatment egg | 0.596 | 0.203 | 34.0 |  |  |  |  |
| Egg 33 | ND | Treatment egg | 0.914 | 0.194 | 21.3 |  |  |  |  |
| Egg 34 | ND | Treatment egg | 0.964 | 0.177 | 18.3 |  |  |  |  |
| Egg 35 | ND | Treatment egg | 0.907 | 0.170 | 18.7 |  |  |  |  |
| Egg 36 | ND | Treatment egg | 0.757 | 0.156 | 20.6 |  |  |  |  |
| Egg 37 | ND | Treatment egg | 0.897 | 0.170 | 19.0 |  |  |  |  |
| Egg 38 | TR | Treatment egg | 0.948 | 0.220 | 23.2 |  |  |  |  |
| Egg 39 | 0.154 | Treatment egg | 0.854 | 0.215 | 25.2 |  |  | 0.718 | 718.4 |
| Egg 40 | TR | Treatment egg | 0.929 | 0.223 | 24.0 |  |  |  |  |
| Egg 41 | ND | Treatment egg | 1.001 | 0.215 | 21.5 |  |  |  |  |
| Egg 42 | ND | Treatment egg | 0.882 | 0.186 | 21.0 |  |  |  |  |
| Egg 43 | 0.832 | Treatment egg | 0.935 | 0.174 | 18.6 |  |  | 4.781 | 4780.6 |
| Egg 44 | ND | Treatment egg | 0.799 | 0.148 | 18.5 |  |  |  |  |
| Egg 45 | ND | Treatment egg | 1.151 | 0.223 | 19.3 |  |  |  |  |
| Egg 46 | 0.165 | Treatment egg | 1.103 | 0.206 | 18.7 |  |  | 0.798 | 798.0 |
| Egg 47 | 0.224 | Treatment egg | 0.884 | 0.183 | 20.7 |  |  | 1.224 | 1224.5 |
| Egg 48 | 0.175 | Treatment egg | 0.661 | 0.148 | 22.4 |  |  | 1.179 | 1179.2 |
| Egg 49 | ND | Treatment egg | 0.878 | 0.188 | 21.4 |  |  |  |  |
|  |  | **Average** | **0.766** | **0.166** | **21.7%** | **85.5%** |  | **1.566** | **1565.6** |
